# Abscisic Acid as an Internal Integrator of Multiple Physiological Processes Modulates Leaf Senescence Onset in *Arabidopsis thaliana*

**DOI:** 10.3389/fpls.2016.00181

**Published:** 2016-02-19

**Authors:** Yuwei Song, Fuyou Xiang, Guozeng Zhang, Yuchen Miao, Chen Miao, Chun-Peng Song

**Affiliations:** ^1^State Key Laboratory of Cotton Biology, Department of Biology, Institute of Plant Stress Biology, Henan UniversityKaifeng, China; ^2^Department of Life Science and Technology, School of Life Science and Technology, Nanyang Normal UniversityNanyang, China

**Keywords:** abscisic acid, leaf senescence, chlorophyll fluorescence, guard cell, cytosolic calcium

## Abstract

Many studies have shown that exogenous abscisic acid (ABA) promotes leaf abscission and senescence. However, owing to a lack of genetic evidence, ABA function in plant senescence has not been clearly defined. Here, two-leaf early-senescence mutants (*eas*) that were screened by chlorophyll fluorescence imaging and named *eas1-1* and *eas1-2* showed high photosynthetic capacity in the early stage of plant growth compared with the wild type. Gene mapping showed that *eas1-1* and *eas1-2* are two novel *ABA2* allelic mutants. Under unstressed conditions, the *eas1* mutations caused plant dwarf, early germination, larger stomatal apertures, and early leaf senescence compared with those of the wild type. Flow cytometry assays showed that the cell apoptosis rate in *eas1* mutant leaves was higher than that of the wild type after day 30. A significant increase in the transcript levels of several senescence-associated genes, especially *SAG12*, was observed in *eas1* mutant plants in the early stage of plant growth. More importantly, ABA-activated calcium channel activity in plasma membrane and induced the increase of cytoplasmic calcium concentration in guard cells are suppressed due to the mutation of *EAS1*. In contrast, the *eas1* mutants lost chlorophyll and ion leakage significant faster than in the wild type under treatment with calcium channel blocker. Hence, our results indicate that endogenous ABA level is an important factor controlling the onset of leaf senescence through Ca^2+^ signaling.

## Introduction

Leaf senescence, involving photosynthesis cessation, degradation of macromolecules, and increase of reactive oxygen species (ROS), as well as contributing to the mobilization of nutrients from old leaves to growing or storage tissues, is regulated by various external and internal factors. In line with this, leaf senescence initiation is affected by many such factors, such as the age of the plant, plant hormones, ROS, transcription factors, protein kinases, nutrient limitation, and drought (Fischer, 2012; Koyama, 2014).

Earlier studies have documented the important role of the phytohormone abscisic acid (ABA) in the regulation of leaf senescence. It has long been considered that ABA accelerates leaf senescence because exogenously applied ABA was shown to promote leaf senescence (Gepstein and Thimann, 1980; Pourtau et al., 2004; Raab et al., 2009; Lee et al., 2011) and endogenous ABA levels have been found to be increased during leaf senescence in many plants (Gepstein and Thimann, 1980; Leon-Kloosterziel et al., 1996; Cheng et al., 2002; He et al., 2005; Breeze et al., 2011; Yang et al., 2014). More importantly, both the upregulation of genes associated with ABA signaling and a dramatic increase in endogenous ABA levels can be observed in many plants during leaf senescence (Tan et al., 2003). Exogenous ABA can induce the expression of many senescence-associated genes (Parkash et al., 2014). In addition, the molecular mechanistic evidence for a positive regulatory role of ABA in senescence comes from functional analyses of receptor-like kinase 1 (RPK1; Lee et al., 2011). RPK1 is a membrane-bound leucine-rich repeat receptor-like kinase that acts as an upstream component of ABA signaling, whose expression was found to increase in an ABA-dependent manner throughout the progression of leaf senescence. Moreover, leaf senescence was accelerated in transgenic plants overexpressing RPK1 and ABA-induced senescence was delayed in *rpk1* mutant plants, suggesting that RPK1 has a role in promoting leaf senescence.

Some studies have shown that ABA inhibits the senescence of cucumber plants grown under low-nitrogen conditions (Oka et al., 2012) and ABA-deficient mutants showed accelerated senescence on glucose-containing medium (Pourtau et al., 2004). In tomato, maize, and Arabidopsis, ABA could maintain shoot growth by inhibiting ethylene production (Sharp, 2002). SAG113 is a PP2C protein phosphatase that acts as a negative regulator of stomatal movement and water loss during leaf senescence (Zhang and Gan, 2012; Zhang et al., 2012). *SAG113* is expressed in senescencing leaves and induced by application of ABA. Leaf senescence was found to be delayed in a *sag113* knockout mutant line (Zhang and Gan, 2012; Zhang et al., 2012). Therefore, the role of ABA in the onset of leaf senescence remains unclear.

It has been reported that several *abscisic acid-dificient* 2 (*aba2*) alleles, as well as other ABA biosynthesis mutants including *aba1, aba3, abscisic aldehyde oxidase 3* (*aao3*), *9- cis-epoxycarotenoid dioxygenase 3* (*nced3*) have already been isolated and identified by screening *Arabidopsis* mutants (Leon-Kloosterziel et al., 1996; Leung and Giraudat, 1998; Laby et al., 2000; Rook et al., 2001; Cheng et al., 2002; González-Guzmán et al., 2002; Finkelstein, 2013). These studies are mainly focused on stomatal regulation, developmental processes, and stress responses. However, little is known whether ABA specifically modulates leaf senescence. Recent studies showed that an *Arabidopsis* NAC-LIKE, ACTIVATED BY AP3/PI (NAP) transcription factor promotes chlorophyll degradation by enhancing transcription of ABSCISIC ALDEHYDE OXIDASE3 (AAO3), which leads to increased levels of the senescence-inducing hormone ABA (Yang et al., 2014).

In this work, we used chlorophyll fluorescence imaging to isolate two early-senescence *Arabidopsis* mutants (*eas1-1* and *eas1-2*) and performed further studies that showed that they are novel *aba2* alleles. Compared with the wild type, the *eas1* mutants display multiple phenotypes including early germination, larger stomatal aperture, insensitivity to stresses, more chloroplasts in mesophyll cells, higher chlorophyll fluorescence during the early stage of plant growth, and early leaf senescence. Meanwhile, many senescence-associated genes were found to be strongly up-regulated in the *eas1* mutants during the early stage of plant growth. Furthermore, [Ca^2+^]_cyt_ levels and calcium channel activity of *eas1* mutant guard cells were significantly lower than those of the wild type. These results revealed that the internal ABA level is involved in the control of senescence onset.

## Materials and methods

### Plant growth conditions and isolation of mutants

*Arabidopsis thaliana* plants used were in the C24 and the Columbia 0 background. Approximately 50,000 M1 seeds of the C24 ecotype were mutagenized by treatment with 0.4% EMS solution for 8 h. M2 seeds were obtained by self-fertilization of the M1 plants. Surface-sterilized seeds were plated in MS medium containing 3% (w/v) sucrose and 0.8% (w/v) agar and, after 5–7 days, seedlings were transplanted into pots containing a mixture of forest soil:vermiculite (3:1). The potted plants were kept under a cycle of 16 h light/8 h dark and a relative humidity of about 50–70% in a growth room at 20 ± 2°C. The seedlings were used for mapping the *EAS1* gene. The mutant plants were back-crossed twice to C24. The descendants of single progeny derived from each backcross were used for all experiments.

### Chlorophyll measurements and stress treatment

Leaves 4 and 5 were detached from plants under normal or stressed conditions. Total chlorophyll was extracted in ethanol and measured spectrophotometrically (He and Gan, 2002). To determine leaf senescence phenotype of *eas1* and wild-type plants under osmotic and oxidative stresses, 20-days-old leaves were floated on water or water containing 10 mM H_2_O_2_ or 500 mM mannitol in petri dishes under normal condition as described in the figure legends.

### Dark treatment

Seedlings grown 20 days after sowing in soil were placed in a closed opaque box in a growth room at 20 ± 2°C. To ensure that the box is not translucent, box was wrapped with aluminum foil. Pictures were taken after 2, 4, 6, 8, and 10 days as indicated in the figure legends.

### Measurements of ion leakage, total DNA content, and protein extraction

Ion leakage and total DNA content in the sixth rosette leaves grown for 25 days under osmotic and oxidative stresses. For measuring ion leakage, leaf samples were immersed into deionised water, shaken in a 25°C water bath for 30 min, and the conductivity was measured using an electrical conductivity meter (B-173, Horiba, Kyoto, Japan). Samples were boiled for 10 min before total conductivity was determined. The conductivity was expressed as the percentage of the initial conductivity versus the total conductivity (Jing et al., 2002). Total DNA content was measured by densitometry method. Leaf total proteins were extracted from 250 mg FW of frozen leaf tissue at 4°C with 2 ml of 100 mM potassium phosphate buffer, pH 7.5. The homogenate was centrifuged (2000 g, 4°C, 5 min) and supernatant was collected.

### ABA quantification

Fresh leaf samples (usually 1 g) was used for ABA content determination assay. Fully expanded leaflets immediately immersed in liquid N_2_ and then stored at −20°C before being used for ABA content determination. ABA was extracted and measured using enzyme-linked immunosorbent assay (ELISA).ELISA kits were purchased from China Agriculture University (China). The assays were performed according to the instructions given by the manufacturer.

### Chlorophyll fluorescence imaging and photosynthetic parameters

Images of chlorophyll fluorescence were obtained as described by Barbagallo et al. (2003) using a CF Imager (Technologica Ltd.,Colchester, UK). Seedlings were adapted to the dark for 30 min before minimal fluorescence (Fo) was measured using a weak measuring pulse. Then, maximal fluorescence (Fm) was measured during 800-ms exposure to a saturating pulse having a photon flux density (PFD) of 4800 μmol m^−2^s^−1^. Plants were then exposed to an actinic PFD of 300 μmol m^−2^s^−1^ for 15 min and steady-state F′ was continuously monitored, while Fm′ (maximum fluorescence in the light) was measured at 5-min intervals by applying saturating light pulses. This was repeated at a PFD of 500 μmol m^−2^s^−1^. Fv/Fm, maximum quantum efficiency of PSII photochemistry.

### Genetic analysis and map-based cloning of the *EAS1* gene

Backcrosses of *eas1* mutants to the wild type and intercrosses among *eas1* mutants, as well as those of *eas1* with *aba* mutants, were performed by transferring pollen to the stigmas of emasculated flowers. The mapping population was generated by crossing *eas1* (C24) to the Col-0 wild type. From the F_2_ generation, 800 homozygous *eas1* individuals were isolated. Genomic cDNA of the young seedling was extracted individually to perform PCR using simple sequence length polymorphism (SSLP) markers to identify recombinants, as described previously (Cheng et al., 2002). Fine mapping was performed by designing new indel markers. The primers were synthesized based on bacterial artificial chromosome (BAC) DNA sequences and tested by PCR using DNA isolated from three ecotypes. *eas1* was found to be linked to the SSLP marker nga280 on the long arm of chromosome I. Thus, SSLP markers were developed based on the sequences of the BAC clones F5F19, F6D8, F12M16, F15I1, T15A14, F16N3, and F7F22.

### Real-time RT-PCR

Total RNA was extracted with TRIzol reagent (Ambion) from leaves 6 and 7 under different conditions and digested with RNase-free DNase I; it was then used for real-time RT-PCR, employing oligo (dT) primers with M-MLV (Promega) in a 30-μL reaction, in accordance with the manufacturer's instructions. The cDNA was used for quantitative real-time PCR amplification. One microliter of the RT reaction was used as a template to determine the levels of transcripts of the tested genes using a PTC-200 DNA Engine Cycler with a Chromo 4 Detector in 25-μL reactions. The levels of actin is described as the control, and the values given are expressed as the ratios to the values in the wild type. Three biological replications were performed for each experiment. The values shown represent averages of triplicate assays for each RT sample. PCR conditions were as follows: 5 min at 95°C (one cycle), and 30 s at 95°C, 30 s at 55–60°C, and 60 s at 72°C (40 cycles). The primers for real-time PCR are shown in Table S1.

### Thermal imaging

A ThermaCAMSC3000-equipped quantum-well infrared photodetector was used as it provides image resolution of 320 × 240 pixels and is responsive to a broad dynamic range, with extraordinary long-wave (8–9 μm) imaging performance. The specified temperature resolution was below 0.03°C at room temperature. The camera was mounted vertically at ~35–45 cm above the leaf canopy for observations, and was connected to a color monitor to facilitate visualization of individual plants. Digitally stored 14-bit images, live IR video, or real-time high-speed dynamic events were analyzed.

### Electrophysiological assays and data acquisition

*Arabidopsis* guard cell protoplasts of leave 5 were isolated as described previously (Tallman, 2006; Zhang et al., 2008). The whole-cell voltage-clamp or single-channel currents of *Arabidopsis* guard cells were recorded with an EPC-9 amplifier (Heka Instruments), as described previously (Bai et al., 2009). Pipettes were pulled with a vertical puller (Narishige, Japan) modified for two-stage pulls. Data were analyzed using PULSEFIT 8.7 software. Standard solutions for Ca^2+^ measurements were used, including 10 mM BaCl_2_, 0.1 mM DTT, 10 mM MES-Tris (pH 5.6) in a bath, and 100 mM BaCl_2_, 0.1 mM DTT, 4 mM EGTA, and 10 mM HEPES-Tris (pH 7.1) in a pipette. ABA was freshly added to bath solutions at the indicated concentrations. For ABA-activated Ca^2+^ current measurements, 50 μM ABA was added to the standard pipette solution. Osmolalities of pipette and bath solutions were adjusted to 510 and 490 mM kg^−1^, respectively, using *D*-sorbitol (Sangon, China).

### Flow cytometric analysis

Analyses were performed on three Cytomics FC500 flow cytometers (Beckman-Coulter, Villepinte, France). To limit background noise from dust and crystals, all three instruments were operated using 0.22-μm filtered sheath fluid (Isoflow™; Beckman-Coulter). CXP ACQUISITION and CXP ANALYSIS software packages (Beckman-Coulter) were used for data acquisition and analysis, respectively. *Arabidopsis* protoplasts of leave 5 were immersed in 5 μM FDA (Sigma; in MES buffer, pH 6.1) for 20 min at room temperature in the dark, and then washed three times with MES buffer (pH 6.1). Cells were stained with Annexin V using the Annexin V-FITC fluorescence detection kit (BD Biosciences, San Jose, CA, USA), in accordance with the manufacturer's instructions. Briefly, cells cultured on cover slips, and then washed twice with PBS. The slides were examined and photographed with a Nikon Eclipse TE 2000 U motorized inverted microscope (Nikon Corp., Tokyo, Japan). The apoptotic index was calculated as the percentage of cells stained positive for Annexin V. A total of 100 cells were counted in each experimental group in three independent experiments and results arethe mean proportion of apoptotic cells in sixscanning electron micrographs.

### Ca^2+^ measurements of the seedlings by Aq bioluminescence and calibration of calcium measurements

Ca^2+^ measurements of wild-type and *eas1-1* mutant seedlings by Aq luminescence were carried out according to the method of Bai et al. (2009). Seven-days-old seedlings were incubated in distilled water containing 2.5 μM coelenterazine (Promega) overnight in the dark at room temperature. A seedling was put into a cuvette with 100 μL of distilled water for 1–2 h in the dark, and then the cuvette was placed inside a TD20/20n digital luminometer (Turner Biosystems). Luminescence was recorded after counting for 20 s, the different reagents were added to the cuvette and the luminescence was measured. At the end of each experiment, the remaining Aq was discharged by the addition of an equal volume of 2 M CaCl_2_ and 20 % ethanol. Luminescence values were converted to the corresponding calcium concentrations. Ten seedlings were used in each experiment.

### Statistical analyses

All experiments were repeated at least three times. To determine significant differences among different lines or different treatments, all the data were analyzed by Dunnett's test using SPSS16.0 software.

## Results

### Leaves of *Eas1* mutant plants display early-senescence phenotypes

Chlorophyll content and photochemical efficiency are well-established senescence parameters and convenient markers, which can be used for assaying leaf senescence (Oh et al., 1997; Woo et al., 2001). To obtain further insights into the role of photosynthesis in leaf senescence, we developed a novel genetic screen for *Arabidopsis* mutants with altered photochemical efficiency during leaf development. This approach uses the ratio of variable (Fv) to maximal (Fm) fluorescence, which represents the quantum efficiency of PSII reaction centers. Fv/Fm can be measured continuously and nondestructively using chlorophyll fluorescence imaging, which provides a convenient indicator of the photosynthesis during leaf development and senescence (Barbagallo et al., 2003; Rolfe and Scholes, 2010; Harbinson et al., 2012). We used chlorophyll fluorescence imaging to screen for *Arabidopsis* mutants that displayed an increased or reduced Fv to Fm ratio during leaf development, in which photosynthesis efficiency thus appeared to be altered (Harbinson et al., 2012). One group of mutants that exhibited a clear increase in Fv/Fm at day 20 after planting was isolated. Two allelic mutants, designated *eas1-1* and *eas1-2* (*early senescence 1-1 and -2*), showing increased photochemical efficiency and early-aging syndrome throughout the whole of their development, were chosen for detailed characterization.

**Figure 1:**
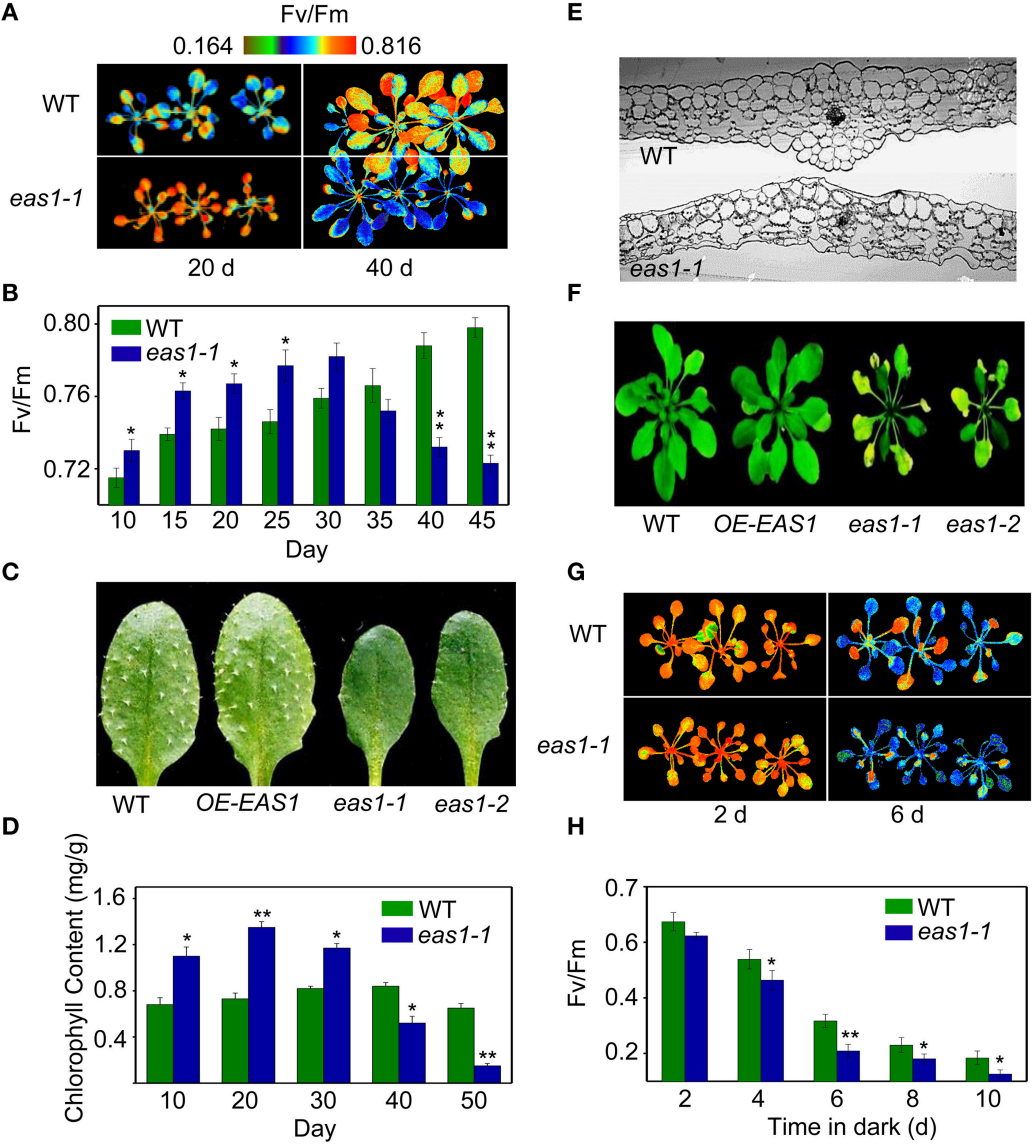
**The ***eas1*** mutation accelerates leaf senescence in ***Arabidopsis thaliana*****. **(A)** Images of Fv/Fm of wild-type and *eas1-1* mutant leaves on the 20th and 40th day under unstressed conditions in soil. **(B)** Fv/Fm values depend on different growth stages in wild-type and *eas1-1* mutant plants. **(C)** Twenty-five-days-old leaves of wild-type and *eas1-1* mutant plants. The sixth rosette leaves are shown. **(D)** Determination of chlorophyll contents of wild-type and *eas1-1* mutant leaves at different stages of plant growth. **(E)** Twenty-five-days-old leaf cross-section anatomy of wild-type and *eas1-1* mutant plants. **(F)** Thirty-five-days-old natural aging of the leaf rosettes of wild-type, overexpression of *EAS1* (OE-EAS1), and *eas1* mutant plants grown under unstressed conditions in soil. The flowers and stems of *eas1* plants were removed. **(G)** Images of Fv/Fm of 20-days-old wild-type and *eas1-1* mutant leaves under dark treatment for 2 and 6 days. **(H)** Time-dependence of Fv/Fm values in wild-type and *eas1-1* mutant plants in dark treatment. Three experiments were performed with similar results. Error bars indicate standard deviations, while asterisks indicate significant differences from wild-type plants under Student's test (^*^p < 0.05, ^**^p < 0.01).

Fv/Fm of *eas1-1* plants was significantly higher than that of the wild type before approximately day 30, whilst it declined from day 30 in *eas1-1* plants (Figures 1A,B). The dark-green leaf phenotype and high chlorophyll content in *eas1-1* mutant plants are also consistent with their high photosynthetic efficiency in the early growth stage (Figures 1C,D). Moreover, the *eas1* mutant appears to have smaller or no trichomes (Figure 1C). Twenty-days-old leaf cross-section anatomy showed more chloroplasts in mesophyll cells in *eas1-1* mutant plants than in wild-type plants (Figure 1E). The pattern of change of Fv/Fm was consistent with the change of chlorophyll content throughout the whole of leaf development (Figure 1C).

To determine whether *EAS1* is involved in the regulation of senescence, we observed the aging syndrome in *eas1* and wild-type plants throughout the whole of leaf development. Wild-type plants exhibited a consistent increase in Fv/Fm before day 45, while *eas1* plants displayed enhanced quantum efficiency of PSII at day 30, which rapidly decreased thereafter (Figure 1B). Consistent with this, the aging syndrome of *eas1* mutants, including leaf yellowing and rosette bolting, appeared early compared with that of the wild type plants (Figure 1F; Figure S1). Furthermore, under unstressed conditions, the *eas1* mutant plants displayed accelerated leaf senescence in soil and Murashige and Skoog (MS) medium (Figure 1F; Figures S1A–C).

Map-based cloning and sequencing showed that the *eas1-1* and *eas1-2* missense mutations were generated in the second exon of the At1g52340 gene and the 190 glutamic acid and the 265 glycine were replaced by lysine and arginine in *eas1-1* and *eas1-2*, respectively (Figure S2A). Surprisingly, this gene is allelic to *ABA2/GIN1/SRE1*, which encodes a short-chain dehydrogenase/reductase (SDR1) that catalyze the final oxidation steps in the conversion of xanthoxin to ABA aldehyde (ABAld; Cheng et al., 2002; González-Guzmán et al., 2002). To further confirm the results of positional cloning, *EAS1* complementation was performed by transforming the missense *eas1-1* mutant with a 7.7-kb *ABA2* genomic clone that includes the 3-kb promoter through *Agrobacterium tumefaciens* (Cheng et al., 2002). Six independent- homozygous T3 lines with clear complementation were isolated. These complementary transgenic plants were restored to wild-type phenotype, such as the premature leaf, the growth defects in cotyledons, and rosettes, the wilting and lack of seed dormancy phenotypes of *eas1*. These results are consistent with the previous obervations (Cheng et al., 2002; González-Guzmán et al., 2002). Moreover, genetic crosses also showed that *eas1* is ABA2 allelic mutants (Table S2).

We have detected ABA content from 20-days-old rosette leaves in *eas1-1* and *eas1-2* plants under unstressed conditions. Indeed, compared with the wild type, ABA content decreased by about 20.8 and 23.5% in *eas1-1* and *eas1-2* plants, respectively (Figure S2B). Seed germination of *eas1* occurred significantly more rapidly than that of the wild type under stress conditions (e.g., mannitol and NaCl) or unstressed conditions (Figures S2C,D). The detached leaves of the *eas1* mutants exhibited excessive transpiration under unstressed conditions (Figures S2E,F).

To further demonstrate whether overexpression of *EAS1* may delay leaf senescence or other unexpected phenotypes, EAS1 overexpression transgenic plants were generated. More than 12 independent transgenic lines were isolated and 8 homozygous transgenic lines presents a consistent phenotype during the whole development. One line (OE-EAS1) were chosen for further study. Confusingly, OE1-EAS1 exhibited no apparent phenotypic differences in aerial structures from the wild type in the early stage (Figures 1C,F; Figure S1C). The young leaves of OE-EAS1 have a slightly larger but not significantly differences in chlorophyll content than that of wild type plants. However, OE-EAS1 could delay leaf senescence and plant flowering (Figure S1A). Leave 5 or 6 of wild-type plants show earlier senescent phenotypes (yellow tip and margin) under unstressed conditions in soil than that of OE-EAS1 plants. The average number of OE-EAS1 rosette leaves before flowering is more 2 leaves than that of wild-type plants.

**Figure 2:**
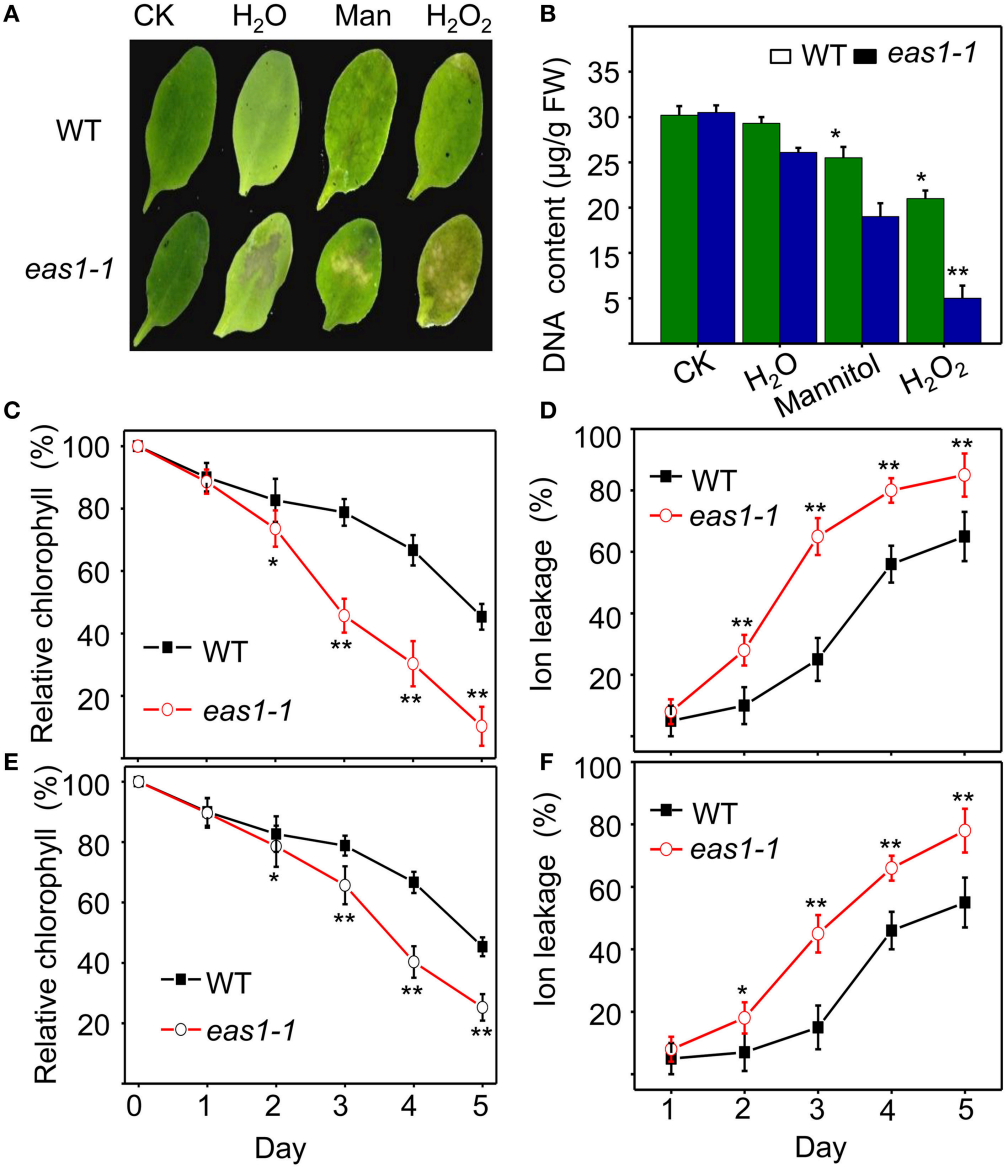
*****EAS1*** mutations accelerate leaf senescence under osmotic and oxidative stresses. (A)** Aging symptoms of detached leaves from wild-type and e*as1-1* mutant plants under different stresses (deionized water, 500 mM mannitol, and 10 mM H_2_O_2_,for 3 days).The sixth rosette leaves grown for 25 days under unstressed conditions in soil are treated. **(B)** DNA content in leaves of different treatments corresponding to panel **(A)**. **(C,D)** Relative chlorophyll contents of wild-type and *eas1-1* leaves under 10 mM H_2_O_2_ **(C)** and 500 mM mannitol **(D)** treatments at different stress times. **(E,F)** Ion leakage of wild-type and *eas1-1* plants at different times under 10 mM H_2_O_2_ **(C)** and 500 mM mannitol **(D)** treatments. Three experiments were performed with similar results. Error bars indicate standard deviations, while asterisks indicate significant differences from wild-type plants under Student's test (^*^p < 0.05, ^**^p < 0.01).

We also examined the senescence syndrome of detached leaves under dark treatment. Upon exposure to dark conditions, the 20-days-old *eas1* leaves displayed more dramatic decreases in Fv/Fm than that of the wild type (Figures 1G,H). Because Fv/Fm of *eas1* plants was significantly higher than that of the wild type (Figures 1A,B) before dark treatment, day 0 control picture is not be shown in Figure 1G. In order to confirm the leaf senescence phenotypes of *eas1* mutant plants, 25-days-old detached leaves were placed under osmotic and oxidative stresses. The results showed that 500 mM mannitol and 10 mM H_2_O_2_ could accelerate leaf senescence of *eas1-1* mutant and wild-type leaves, but the aging symptoms (yellowing and necrotic spots) of *eas1-1* mutant leaves appeared earlier and were more pronounced than those of wild-type leaves (Figure 2A). Relative chlorophyll contents in *eas1-1* mutant leaves were significantly decreased under 10 mM H_2_O_2_ and 500 mM mannitol treatment from the second day of treatment (Figures 2C,D). By contrast, ion leakage in *eas1-1* leaves increased significantly from the third day (Figures 2E,F). The detected level of DNA content was significantly reduced in *eas1-1* mutant leaves (Figure 2B).

### EAS1/ABA2 is involved in regulation of the expression of senescence- associated genes in the early stage of plant growth

**Figure 3:**
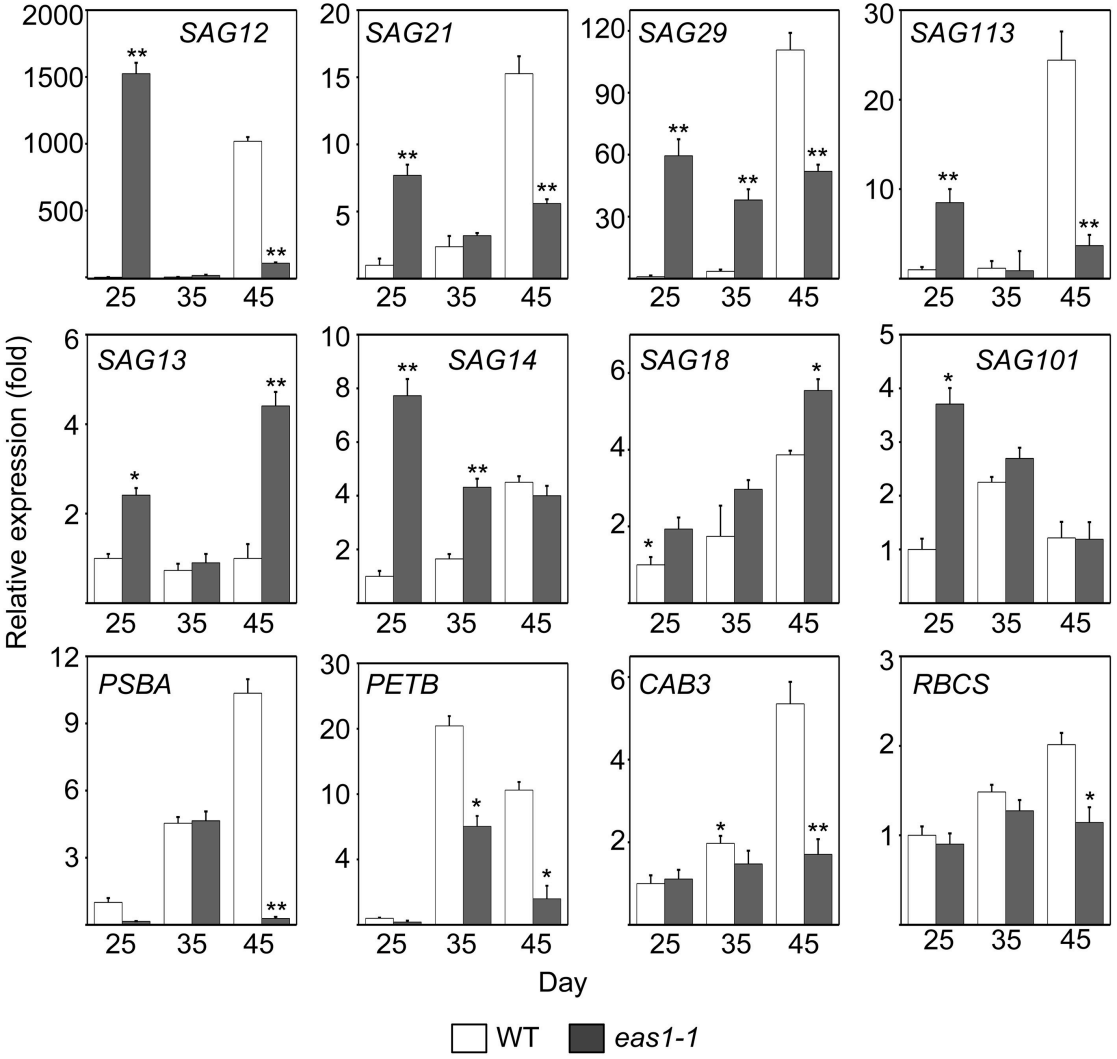
**Expression analysis of SAGs and CRGs in wild-type and ***eas1-1*** mutant leaves for 25, 35, and 45 days under unstressed conditions in soil**. SAGs included *SAG12, SAG13, SAG14, SAG18, SAG20, SAG21, SAG29, SAG101*, and *SAG113*. CRGs included *PSBA, RBCS, CAB3*, and *PETB. Actin* was used as an internal control. Total RNA was isolated from the sixth and seventh true leaves at the indicated time points. Bars indicate standard errors, while asterisks indicate significant differences from wild-type plants under Student's test (^*^p < 0.05, ^**^p < 0.01); three experiments were performed with similar results.

Since the role of ABA in leaf senescence has not been clearly defined, and only circumstantial evidence has been obtained, we further examined the expression of some senescence-associated genes (SAGs) in wild-type and *eas1* mutant plants at different developmental stages. Quantitative reverse transcription PCR (qRT-PCR) was performed using RNA samples from leave 6 and 7 of wild-type and *eas1* mutant plants grown for 25, 35, and 45 days (Figure 3). The *eas1* mutation has been shown to have dramatic effects on the expression of *SAG12* (Noh and Amasino, 1999), *SAG29* (Seo et al., 2011), *SAG113* (Zhang and Gan, 2012), and *SAG101* (He and Gan, 2002) at young and old developmental stages. Compared with the wild type, the expression levels of *SAG12, SAG29, SAG113*, and *SAG21* in leaves of 25-days-old *eas1-1* were increased by 1523-, 59-, 8.5-, and 8-fold, respectively. However, at day 45, the expression of all four genes was significantly inhibited by the mutation (Figure 3). The expression levels of the other *SAGs* (*SAG13, SAG14, SAG18, SAG101*) were also up-regulated from 1.5- to 7.8-fold in *eas1* mutants at day 25. The age-dependent induction of *SAG13, SAG14, SAG18*, and *SAG101* also substantially increased at days 35 and 45.

We also examined the mRNA levels of chloroplast-related genes (CRGs; *CAB3, RBCS, PSBA, PETB*) at 25-, 35-, and 45-d-old *eas1-1* plants (Figure 3). Compared with *eas1-1* mutant plants, the expression levels of almost all CRGs in wild-type plants gradually increased, and *RBCS, PSBA, PETB*, and *CAB3* were significantly up-regulated.

### Mutation of *EAS1* enhances cell apoptosis under leaf senescence

**Figure 4:**
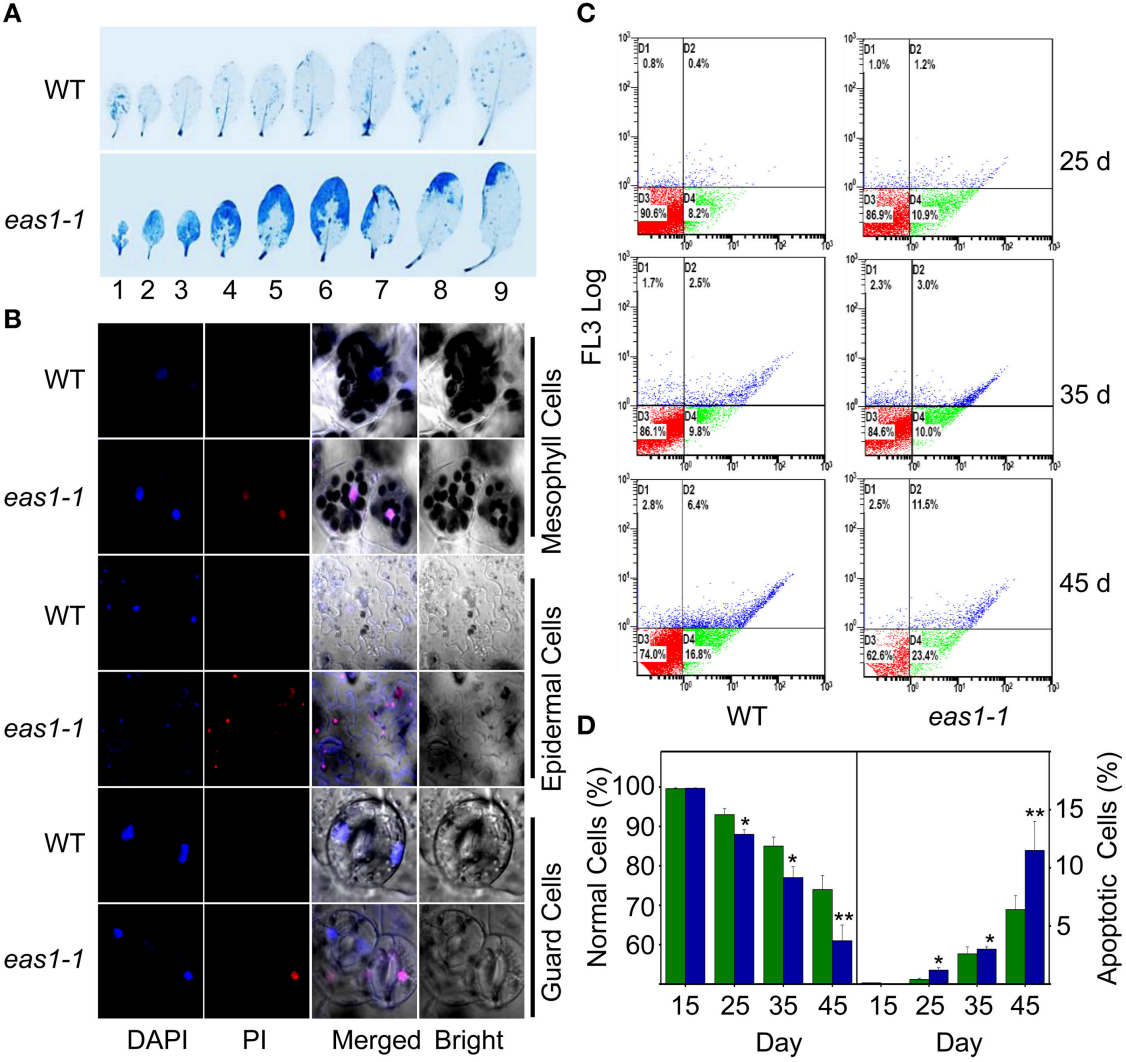
**EAS1 mutation causes earlier apoptosis in leaves of ***eas1-1*** mutant plants than in wild-type plants. (A)** The first to ninth true leaves of wild-type and *eas1-1* mutant plants at 35 days after sowing under unstressed condition in soil. Leaves were stained with trypan blue for the detection of dead cells. **(B)** Estimation of cell membrane integrity by PI staining using confocal images. The sixth leaves of wild-type and *eas1-1* mutant plants at 35 days after sowing were used to isolate mesophyll cells, epidermal cells, and guard cells under unstressed condition in soil. Cells were subjected to 10 μM DAPI and 10 μg/mL PI staining for 0.5 h. **(C)** Quantification of age-dependent cell apoptosis by flow cytometry. Mesophyll protoplasts of sixth leaves were counted by cytometry in wild-type and *eas1-1* mutant plants at 25, 35, and 45 days after sowing under unstressed condition in soil. Cell counts in the four regions D1, D2, D3, and D4 represent the proportions of late-senescent cells, apoptotic cells, normal cells, and early-senescent cells, respectively. **(D)** Normal and apoptotic cell percentages of wild-type and *eas1* mutant protoplasts. The ratio of normal and apoptopic cells were the average of 15 results from three independent experiments. CXP ACQUISITION and CXP ANALYSIS software packages (Beckman-Coulter) were used for data acquisition and analysis, respectively. Bars indicate standard errors, while asterisks indicate significant differences from wild-type plants under Student's test (^*^p < 0.05, ^**^p < 0.01).

It is known that leaf senescence is a programmed event that can be induced by a variety of endogenous factors and environmental cues (Lim et al., 2003; Guo and Gan, 2005; Zhang and Zhou, 2013; Li et al., 2014). To confirm whether *EAS1* mutation could affect the programmed cell death (PCD) in leaf senescence, several fluorescence-based dyes for the measurement of cell death were applied. In the first set of experiments, trypan blue (TB) staining was used for the investigation of cell viability (Lee et al., 2011). When seedlings were stained with TB, a large number of blue patches were observed in leaves of *eas1-1*, but were rarely present in wild-type plants (Figure 4A), indicating that age-dependent cell death is increased in *eas1* mutants during leaf senescence. Consistent with these observations, total protein extracted from *eas1-1* was significantly decreased compared with that of wild-type plants (Figure S3C).

Membrane deterioration is one of the early events during leaf senescence (Leshem et al., 1984). Therefore, we next examined the membrane integrity of protoplasts of leave 6 in wild-type and *eas1-1* plants by using propidium iodide (PI) staining under fluorescence microscopy (Rolny et al., 2011). To test the cell membrane integrity in *eas1-1* and wild-type plants, PI staining was used to estimate cell death. When 35-days-old detached leaves were incubated for 0.5 h, there was significant staining of mesophyll and epidermal tissues in *eas1-1*. However, PI staining was hardly observed in the leaves of wild-type plants (Figure 4B). Furthermore, mesophyll protoplast activity was detected by fluorescein diacetate (FDA) staining (Figure S3A).There was a greater proportion of active mesophyll protoplasts in *eas1-1* mutant plants than in wild-type plants after 35 days (Figure S3B).

For the assessment of cell apoptosis in *eas1* mutant and wild-type plants at different development stages, we stained mesophyll protoplasts with Annexin V-fluorescein isothiocyanate (Annexin V-FITC) and detected apoptotic cells by flow cytometry. After approximately 30 d, the number of normal mesophyll protoplasts in *eas1* mutant plants significantly decreased compared with that in the wild type, while the number of apoptotic cells was increased in *eas1-1* mutant plants (Figures 4C,D). The rates of apoptotic cells in wild-type and *eas1-1* mutant plants were 6.4 and 11.5%, respectively, at day 45, indicating that *EAS1* mutation was associated with a lower survival rate. Together, these results suggest that *EAS1* partially protects cells against senescence-induced PCD.

### ABA signaling, not stomatal behavior, is the causal factor of senescence

The previous series of data establish a general parallel between stomatal aperture size and senescence (Thimann and Satler, 1979a,b; Gepstein and Thimann, 1980), with a strong indication that the stomatal apertures are the causal factor and the effects of stomatal apertures on senescence are actually mediated by the internal concentration of ABA.

**Figure 5:**
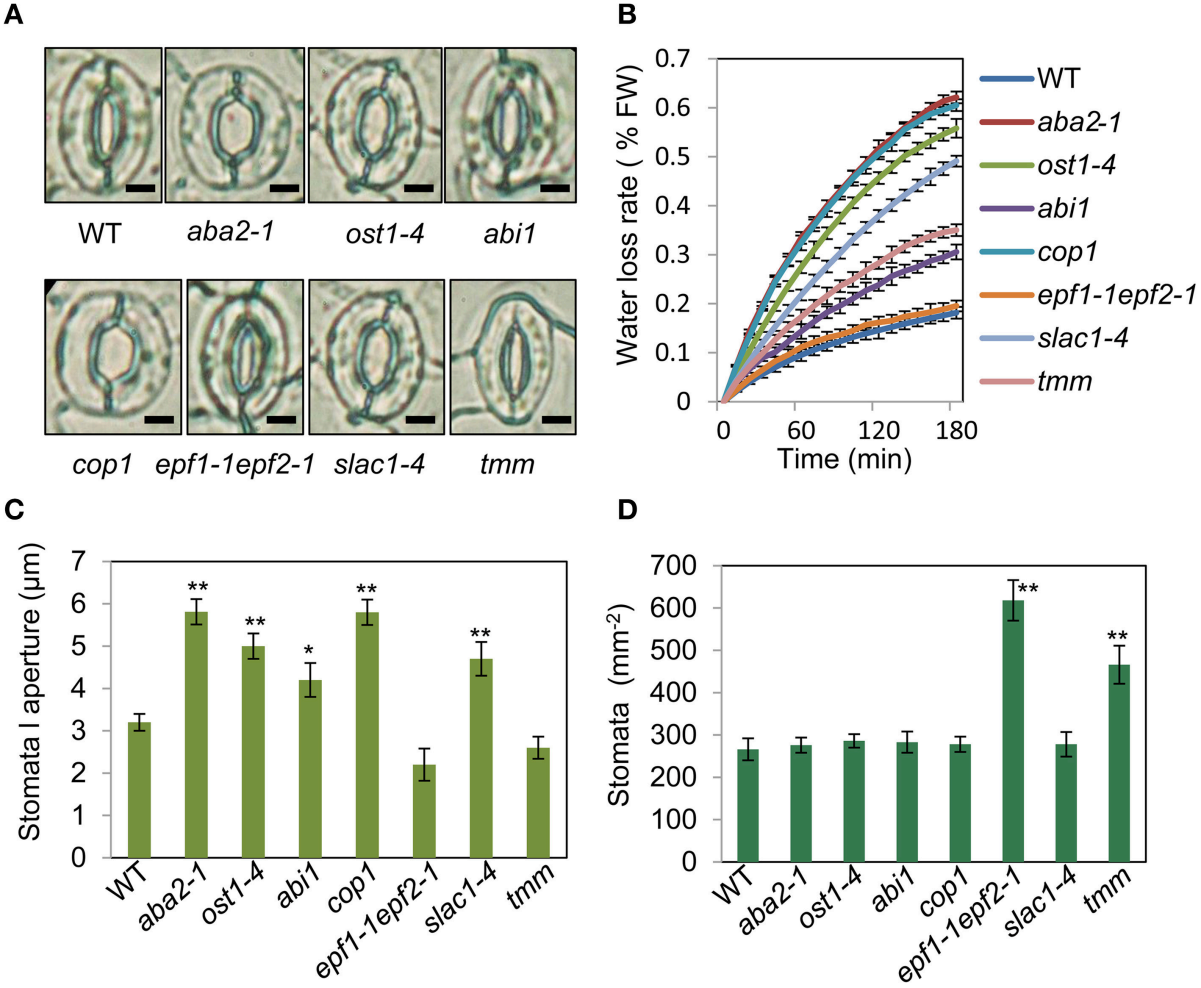
**Analysis of several stomatal mutants in terms of stomatal aperture size and stomatal density. (A)** Representative images of stomata of wild-type and stomatal mutant plants at noon under unstressed conditions in soil. The epidermis of fifth leaf were quickly separated and observed by microscopy. Bar = 10 μm. **(B)** The *aba2-1, cop1, ost1-4, slac1-4, abi1*, and *tmm* mutants lost water more rapidly than the wild-type plants. Water loss from the detached leaves of plants in fresh weight (FW) at day 35. Leaves 5 and 6 were selected for experiments. The rate of water loss from high to low was as follows: *aba2* > *cop1* > *ost1-4* > *slac1-4* > *tmm* > *abi1* > *epf1-1epf2-1* > WT. **(C,D)** Abaxial stomatal densities and stomatal aperture sizes of wild-type plants and stomatal mutants with fifth fully expanded leaves. Bars indicate standard errors, while asterisks indicate significant differences from wild-type and mutant plants under Student's test (^*^p < 0.05, ^**^p < 0.01); three experiments were performed with similar results.

**Figure 6:**
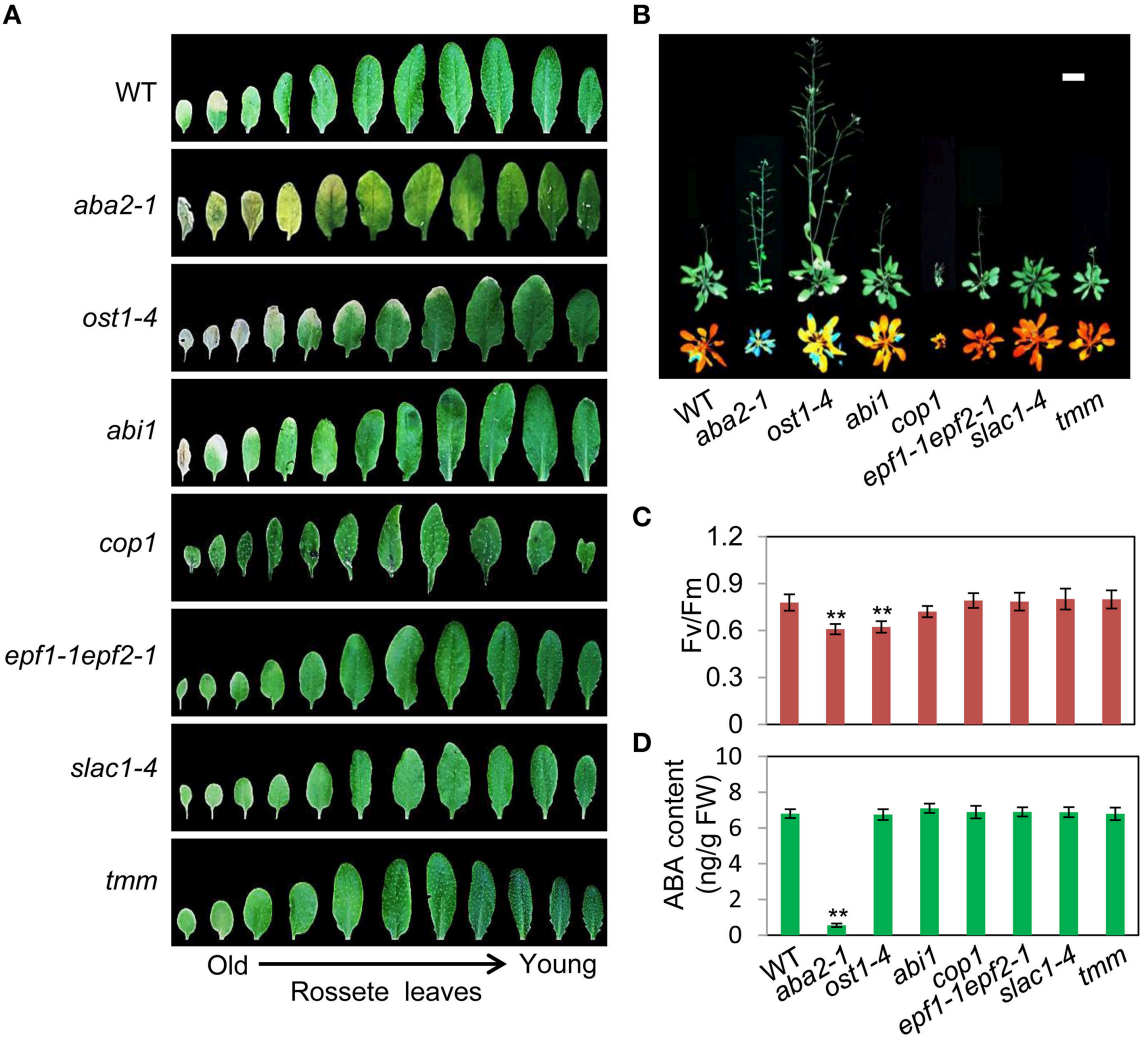
**OST1 mediates ABA regulation of leaf senescence, and stomatal behavior is not involved in this process. (A)** The aging symptoms of rosette leaves at day 45 under unstressed conditions in soil. The first to eleventh rosette leaves are arranged from left to right. In order to highlight the leaf senescence phenotype, images are not to scale enlarge. **(B)** Representative images of wild-type and mutant plants at day 45 under unstressed conditions in soil, upper: bright; lower: chlorophyll fluorescence. The pictures show the aerial parts of plants. Bar = 5 cm. **(C)** Fv/Fm of wild-type and mutant rosette leaves under unstressed conditions in soil at day 45. **(D)** ABA content of the fifth rosette leaf of wild-type and mutant plants under unstressed conditions in soil at day 25. Bars indicate standard errors, while asterisks indicate significant differences from wild-type plants under Student's test (^**^p < 0.01); three experiments were performed with similar results.

To assess the relationship between stomatal apertures and age-dependent leaf senescence, we first measured stomata aperture size and density in several stomatal response and development mutants under unstressed conditions. The results showed that *constitutive photomorphogenic1* (*cop1*; Mao et al., 2005), *slow anion channel-associated 1* (*slac1*; Vahisalu et al., 2008), *open stomata 1* (*ost1*; Mustilli et al., 2002), and *ABA insensitive 1* (*abi1*) showed larger stomatal apertures than that of the wild type (Figures 5A,C). Stomatal openings of the double-mutant *epidermal patterning factor (epf)1-1epf2-1* (Hunt and Gray, 2009) and *too many mouths* (*tmm*; Yang and Sack, 1995) were smaller, but their stomata were present at a higher density than in the wild type (Figures 5A,D). The level of water loss of detached leaves of these mutant plants was higher than that of wild-type plants (Figure 5B). Similar to *aba2-1* mutants*, ost1-4*, and *abi1* mutants displayed earlier leaf senescence than wild-type plants. However, leaf senescence phenotypes of *cop1, tmm*, and *epf11-1 epf2-1* mutants did not differ from those of wild-type plants (Figures 6A,B). The changes of Fv/Fm at different developmental stages displayed similar patterns to those in the observations on leaf senescence (Figure 6C).

To confirm that ABA rather than stomatal behavior modulates the onset of leaf senescence, we determined the ABA content of the above-mentioned stomatal mutants. Except for *aba2-1*, the ABA content of the mutants was similar to that of the wild type (Figure 6D). These results demonstrated that the degree of opening and density of stomata are not necessarily linked to leaf senescence, and ABA signaling is involved in regulation of the onset of leaf senescence.

### Calcium ions are involved in ABA signaling in the regulation of leaf development and senescence

**Figure 7:**
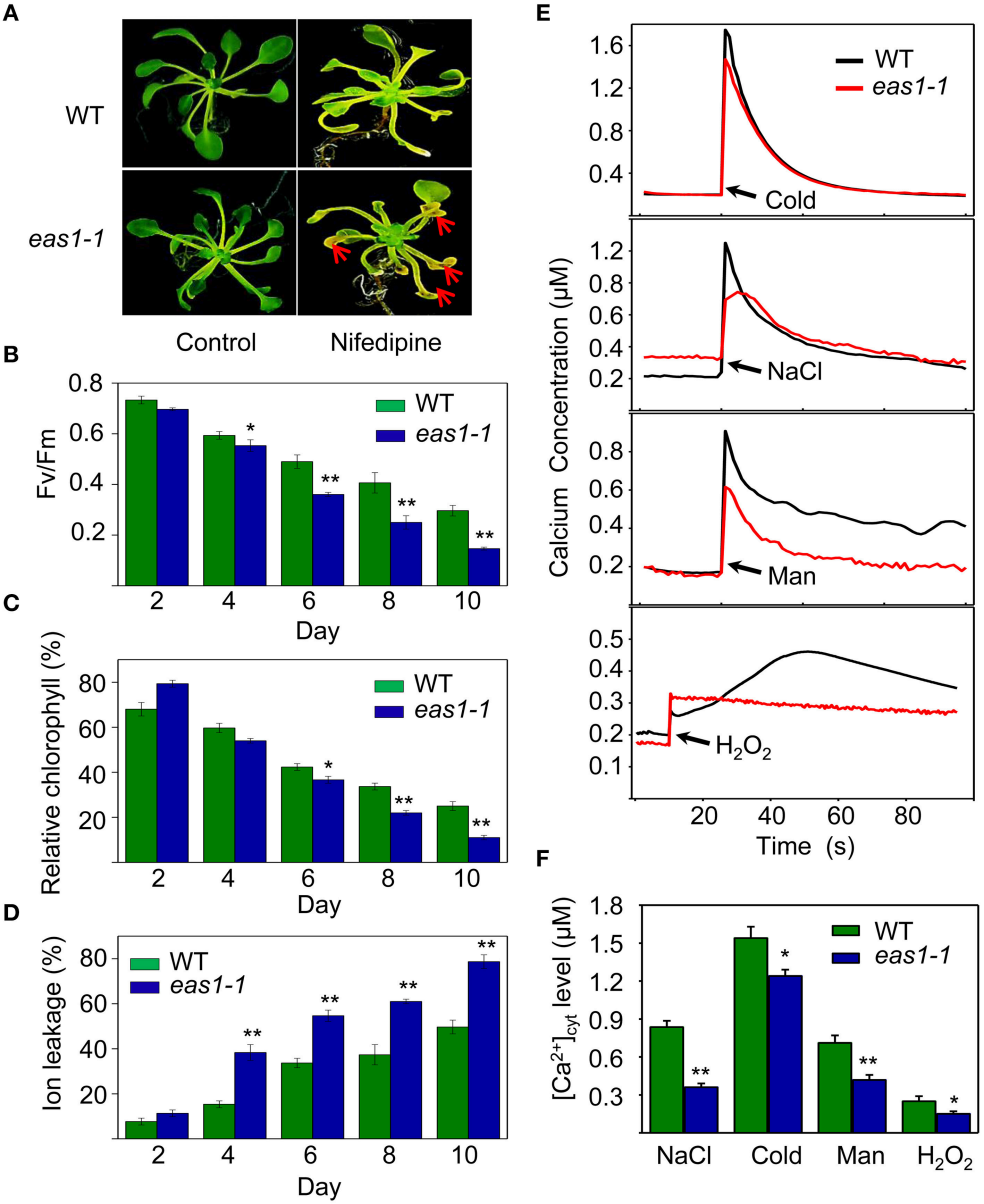
**Calcium deficiency accelerates leaf senescence of ***eas1-1*** mutant plants and analysis of kinetic changes in [Ca^**2+**^]_**cyt**_ in response to several stresses in wild-type and ***eas1-1*** mutant seedlings. (A)** Seedlings grown on MS medium for 5 days were then moved to MS medium supplemented with 200 μM nifedipine for 8 days. Clear aging symptoms are shown by red arrows. **(B)** Time-dependent Fv/Fm values of wild-type and *eas1-1* mutant seedlings on medium supplemented with 200 μM nifedipine. **(C,D)** Relative chlorophyll contents and ion leakage at different times in wild-type and *eas1-1* leaves on medium supplemented with 200 μM nifedipine. **(E)** Elevation of [Ca^2+^]_cyt_ measured by Aq-emitted luminescence in response to cold shock (4°C), 400 mM NaCl, 500 mM mannitol, and 10 mM H_2_O_2_ in 7-days-old wild-type and *eas1-1* mutant seedlings. **(F)** Increased Ca^2+^ concentrations evoked by cold shock (4°C), 400 mM NaCl, 500 mM mannitol, and 10 mM H_2_O_2_ in 7-days-old wild-type and *eas1-1* mutant seedlings. Bars indicate standard errors, while asterisks indicate significant differences from wild-type plants under Student's test (^*^p < 0.05, ^**^p < 0.01); three experiments were performed with similar results.

The transduction of hormonal signals and other environmental stimuli in plant systems is in many instances mediated through the secondary messenger action of Ca^2+^ (Poovaiah and Reddy, 1987), which is involved in the regulation of leaf senescence (Poovaiah and Leopold, 1973; Ma and Berkowitz, 2011). On the basis of our results, we hypothesized that ABA might activate calcium signaling as a means of affecting cell function via membrane deteriorative processes. To test this hypothesis, we examined whether calcium plays a role in the processes by which ABA inhibits senescence. Five-days-old seedlings grown on MS medium were then moved to MS agar plates containing nifedipine, a Ca^2+^ channel blocker. Although nifedipine can accelerate leaf senescence, leaves of *eas1-1* seedlings showed a stronger, more pronounced, and earlier senescent phenotype than those of the wild type, characterized by yellowish apoptotic leaves (Figure 7A). Further analysis showed that Fv/Fm and chlorophyll content decreased more rapidly in 8-days-old leaves of *eas1-1* seedlings than in those of wild-type plants (Figures 7B,C). In contrast, ion leakage was significantly increased and, on the fourth day of treatment, ion leakage of *eas1-1* mutant leaves reached 38.3%, while it was only 15.4% in the wild type (Figure 7D).

It has been reported that the concentration of cytosolic free calcium ([Ca^2+^]_cyt_) play important roles in ABA signaling in plants (Jammes et al., 2011; Cheval et al., 2013). The changes of [Ca^2+^]_cyt_ in wild-type and *eas1-1* mutant seedlings under cold shock, osmotic, and H_2_O_2_ stresses were thus measured using an *Aequorea victoria* (Aq) bioluminescence-based Ca^2+^ imaging method (Bai et al., 2009). As shown in Figure 7E, more significant elevation of [Ca^2+^]_cyt_ was observed in wild-type plants than in *eas1-1* mutant plants. The average values of increased [Ca^2+^]_cyt_ promoted by different stresses in the wild type were 1.25- to 2.63-fold of those in *eas1-1* mutant plants (Figure 7F), which suggested that the *eas1* mutation decreased [Ca^2+^]_cyt_ elevation under cold, NaCl, mannitol, and H_2_O_2_ treatment.

### Effects of ABA on the expression of calcium channel genes and disruption of ABA-activated Ca^2+^ Channel Activity in *eas1-1* guard cells

**Figure 8:**
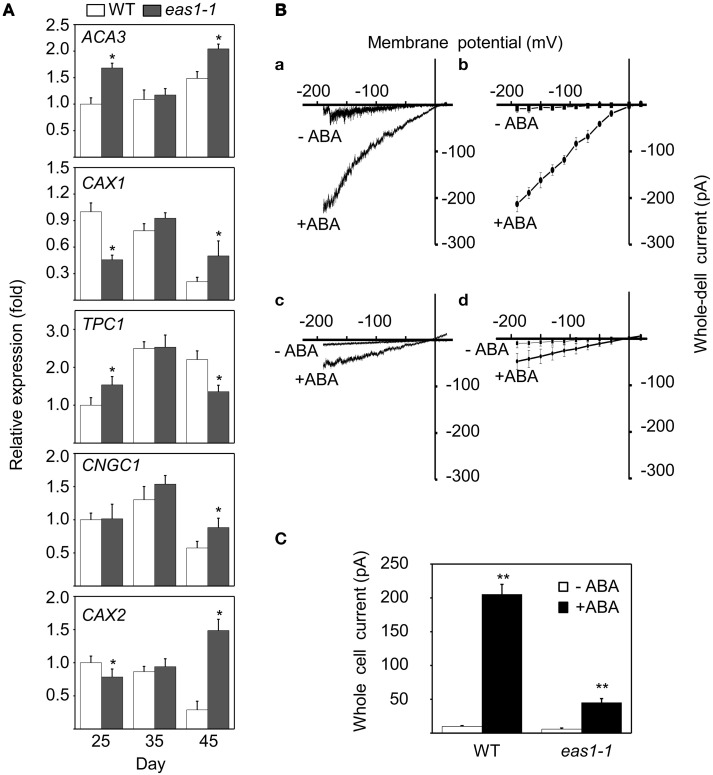
**Expression of several calcium channel genes and changes of Ca^**2+**^ influx currents activated by ABA in guard cells. (A)** mRNA expression levels of *ACA3, CAX1, TPC1, CNGC1*, and *CAX2* in leaves from wild-type and *eas1-1* mutant plants at 25, 35, and 45 days under unstressed conditions in soil. *Actin* was used as an internal standard. Total RNA was isolated from the sixth and seventh true leaves at the indicated time points. **(B) (a,c)** Whole-cell Ca^2+^ influx currents in guard cells of wild-type and *eas1-1* mutant plants with or without 50 μM ABA; **(b,d)** Statistical analysis of Ca^2+^ channel currents in **(a,c)** (*n* = 10). **(C)** Average Ca^2+^ currents at 150 mV in wild-type and *eas1* mutant guard cells with or without 50 μM ABA treatment. Error bars indicate standard deviations, while asterisks indicate significant differences from wild-type plants under Student's test (^*^p < 0.05, ^**^p < 0.01); three experiments were performed with similar results.

In order to explain why basal [Ca^2+^]_cyt_ and increased [Ca^2+^]_cyt_ in response to stresses were lower in *eas1-1* mutant plants than in wild-type plants, the expression levels of several calcium channel genes (*ACA3, CAX1, TPC1, CNGC1*, and *CAX2*) were determined by qRT-PCR. The expression of *CAX1* and *CAX2* was down-regulated, and that of *ACA3* and *TPC1* was slightly up-regulated in *eas1* mutant plants compared with that of wild-type plants at day 25. However, no difference in the expression of these genes was observed at day 35. At day 45, the expression of *CAX2* was apparently up-regulated (approximately seven-fold) and the expression of *TPC1* was down-regulated by ~60% (Figure 8A) in *eas1-1* mutant plants compared with wild-type plants.

To examine whether endogenous ABA was responsible for the activation of Ca^2+^ influx currents, Ca^2+^ influx conductance in wild-type and *eas1-1* mutant plants was monitored by the patch-clamp technique. In whole-cell patch-clamp recording using conditions described previously (Bai et al., 2009), 50 μM ABA markedly evoked influx currents in guard cells of both wild-type and *eas1-1* mutant plants (Figures 8Ba,c). The average values of Ca^2+^ channel currents also confirmed these results (Figures 8Bb,d). In wild-type plants, treatment with ABA significantly induced Ca^2+^ channel activity for inward Ca^2+^currents compared with the control, which rose from <30 pA at time zero to 205 pA (Figure 8C). In contrast, treatment of mutants with ABA had a minimal effect on Ca^2+^channel activity compared with that in controls, with changes no greater than 50 pA being observed (Figure 8C).

## Discussion

### ABA is an inhibitor of leaf senescence

A crucial link between ABA and leaf senescence has yet to be discovered via genetic analysis. In this work, we have established a leaf senescence screening system based on chlorophyll fluorescence and successfully isolated *eas1*mutantsby chlorophyll fluorescence imaging. We were surprised to find that *eas1* is an *aba2* allelic mutant. It has long been known that ABA is a senescence promoter (Mizrahi et al., 1975; Gepstein and Thimann, 1980) and endogenous ABA levels play an important role in the regulation of leaf senescence (Pourtau et al., 2004; Liang et al., 2014; Yang et al., 2014). Our genetic and physiological evidence indicates that *EAS1* mutations rapidly decreased the efficiency of leaf photosynthesis and caused early leaf senescence after day 35 in natural developmental conditions (Figure 1). Experiments on age-dependent PCD in leaves showed more significant and earlier apoptosis in *eas1* plants than in wild-type plants under unstressed conditions (Figure 4). In response to osmotic or oxidative stress, the detached leaves of *eas1* mutant also displayed phenotypes of higher sensitivity and earlier senescence than those of the wild type under dark treatment (Figure 2). Hence, it is suggested that ABA has a clear role in delaying leaf senescence, at least under dark-induced conditions. Recent studies showed that an Arabidopsis *NAC-LIKE, ACTIVATED BY AP3/PI* (NAP) transcription factor promotes chlorophyll degradation by enhancing transcription of *AAO3*, which leads to increased levels of the senescence-inducing ABA (Yang et al., 2014). However, our results clearly show that leaves of *eas1* plants have higher chloroplast density, chlorophyll concentration and appear greener at 25 days. This appears to be a concentration effect due to inhibited growth since the leaves are smaller (Figure 1). These contradictory results may be due to leave age and experimental condition. Moreover, *aao3* mutant seeds display normal seed dormancy (Seo et al., 2000; Finkelstein, 2013). It seems to imply that ABA2/EAS1 and AAO3 may play different roles in the regulation of leaf senescence.

The analysis of *eas1*/*aba2* mutants led us to the idea that ABA function is an age-dependent response in plant development and senescence. It appears that ABA controls both cellular protection activities and senescence activities. The balance between these two activities seems to be important in controlling the progression of leaf senescence and may be adjusted by other senescence-affecting factors such as age. In young plants, ABA is an internal orchestrator that balances the activities that promote morphogenesis and inhibition set of deterioration processes in plant growth and development. By contrast, in old plants (similar to stress conditions), ABA's protective effects decreased and its senescence activity increased. In fact, we found that several SAGs exhibited earlier and stronger expression in the early growth stage of *eas1* mutant leaves than in the wild type. For example, *SAG12*, an *Arabidopsis* gene encoding a cysteine protease, is expressed only in senescent tissues (Noh and Amasino, 1999). *SAG12* expression is specifically activated by developmentally controlled senescence pathways but not by stress- or hormone-controlled pathways (Noh and Amasino, 1999). In contrast, the expression of *SAG12* in *eas1* mutant leaves was approximately 1500-fold higher than that in wild-type plants on day 25. Furthermore, the expression of *SAG29, SAG21*,and *SAG113* was also higher in *eas1* mutant leaves than in the wild type. In addition to these SAGs, other types (*SAG13, SAG14, SAG18, SAG101*) all displayed different degrees of up-regulation in *eas1* mutant plants. Interestingly, we found that the levels of RNA transcribed from most of the SAG genes examined (e.g., *SAG12, 21, 29*, and *113*) in leaves of *eas1* plants were significantly reduced in comparison to those of wild-type plants after day 45 (Figure 3). In addition, there was higher chlorophyll content and Fv/Fm ratio, as well as a significant increase in *eas1-1* plants before day 30. In contrast, these features were significantly attenuated after day 35, which suggested that day 30 is a turning point regarding ABA's function in *A. thaliana*. Over the course of development, the role of ABA decreases, at which time some stress-response genes and senescence-associated genes may start to function and produce senescence syndrome. Therefore, this integrated senescence response provides plants with optimal fitness by incorporating their environmental and endogenous status in a given ecological setting by fine-tuning the initiation timing, progression rate, and nature of leaf senescence.

### ABA is the internal integrator of leaf senescence onset through [Ca^2+^]_cyt_

Leaf senescence is basically governed by the developmental age. However, it is also influenced by various internal and environmental signals that are integrated into the age information. Our data provide genetic, molecular, and physiological evidence supporting the essential function of ABA in the onset of leaf senescence.

Similar to gibberellin and cytokinin (Zwack and Rashotte, 2013; Chen et al., 2014), a low concentration of calcium (0.1–1.0 μM) can delay leaf senescence by suppressing the decreases in chlorophyll and protein content, as well as the increase in hydraulic permeability (Poovaiah and Leopold, 1973). The application of a Ca^2+^ channel blocker hastened the senescence of detached wild-type leaves maintained in the dark, increasing the rate of chlorophyll loss, the expression of a senescence-associated gene, and lipid peroxidation (Ma and Berkowitz, 2011). Moreover, a calmodulin (CaM) antagonist enhanced the accumulation of the transcripts of senescence genes in detached leaves and CaM signaling could attenuate leaf senescence by inhibiting the expression of such genes (Fujiki et al., 2005). Similar results were obtained here in that *eas1* mutant plants displayed early senescence of leaves upon calcium blocker treatment (Figures 7A–D). The elevation in [Ca^2+^]_cyt_ was inhibited in *eas1-1* mutant plants in response to multiple stresses (Figures 7E,F). The expression of calcium channel genes was enhanced and ABA-activated Ca^2+^ channel activity was disrupted in *eas1-1* guard cells (Figure 8). Thus, it is possible that endogenous ABA-induced transient increase in [Ca^2+^]_cyt_ is an important component of early leaf senescence.

Earlier physiological observations that light-induced stomatal opening suppressed oat leaf senescence and stomatal closure accelerated or promoted senescence indicated that opening and closing of leaf stomata is the initial factor associated with senescence (Thimann and Satler, 1979a,b; Gepstein and Thimann, 1980). Mutants that are defective in ABA synthesis and stomatal response provide effective tools to dissect the relationship between stomatal behavior and senescence onset. When the stomata open due to ABA deficiency, the release of blue signal in *cop1* mutant, or lowered ion transport activity, the results from these stomatal mutants are not the same in terms of leaf senescence: only the ABA-deficient mutants showed the promotion of senescence; Conversely, the *cop1* mutant did not display early-senescence syndrome, although it showed larger stomatal apertures compared with wild-type plants (Figures 5, 6). In addition, transgenic *Arabidopsis* plants overexpressing RAP2.6L showed delayed water logging-induced early senescence by an increase of ABA content, stomatal closure, and antioxidant enzyme activity (Liu et al., 2012). Keeping ABA at the basal level is very important for plant development or stomatal regulation (Cheng et al., 2002). These results indicate that there is a close positive correlation between stomatal aperture size and plant senescence, but leaf senescence depends on the endogenous ABA level. This counters the assertion that stomata aperture size is the initial factor of senescence, and supports the fundamental role of the endogenous level of ABA in leaf senescence onset.

**Figure 9:**
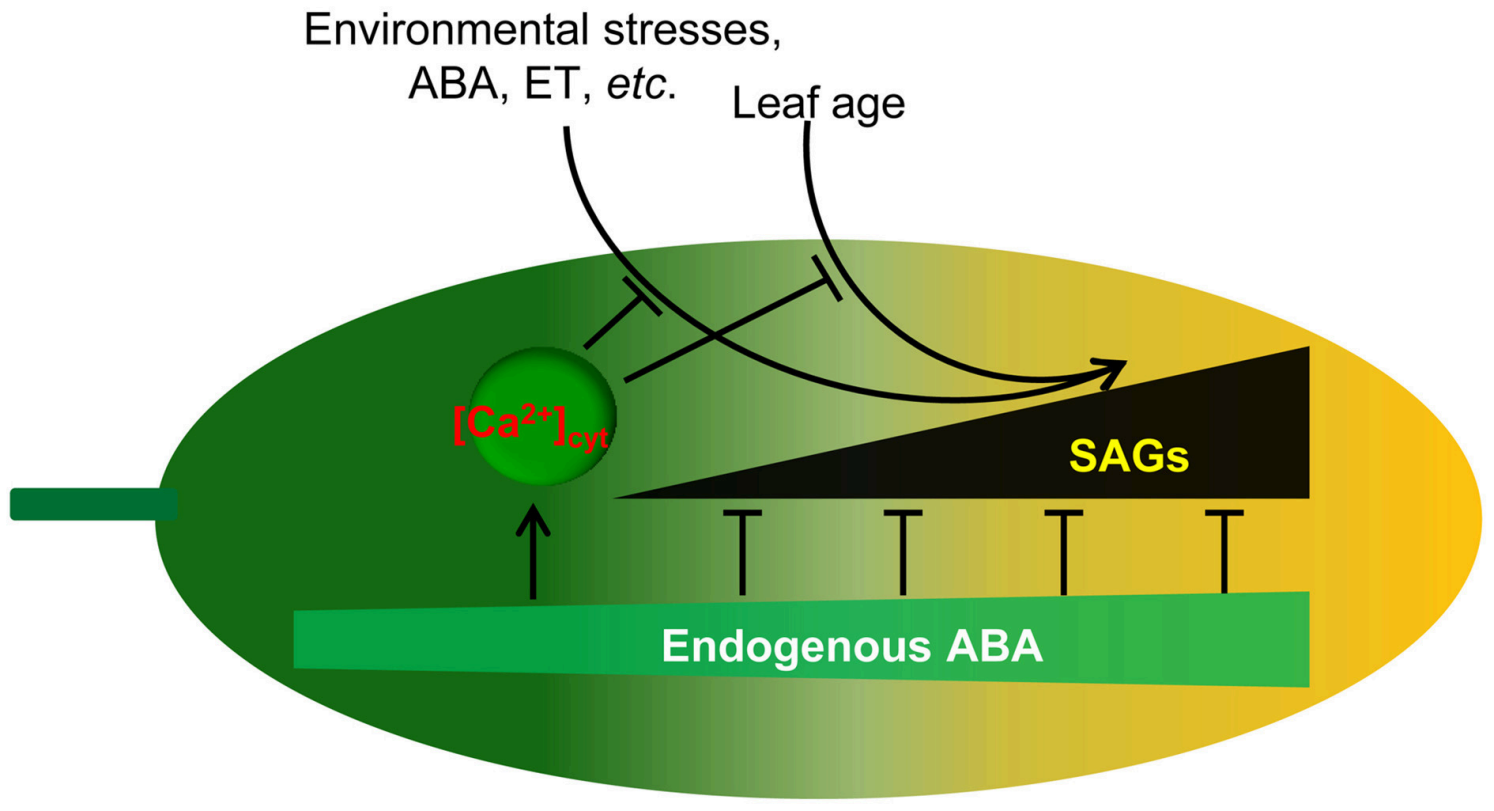
**Proposed model for endogenous ABA function in leaf senescence**. Leaf age or environmental stresses trigger the onset of senescence through the promotion of SAG expression. Endogenous ABA not only promotes plant growth and development but also inhibits leaf senescence onset through the inhibition of SAG expression. Meanwhile, endogenous ABA content increases with environmental stresses and leaf age. [Ca^2+^]_cyt_ is involved in the regulation of this process. Thus, ABA appears to act as a key regulator linking internal and external factors and leaf senescence.

In summary, we suggest that ABA functions in development and senescence by orchestrating gene expression and the accumulation of physiological changes, which is similar to the theory of the yin-yang balance in traditional Chinese medicine (Figure 9). Keeping yin-yang in harmony is akin to attaining a homeostatic state (Ou et al., 2003), and the imbalance of yin-yang has been considered to be the cause of all disease. Similarly, plants enter senescence in yin, in which the role of ABA in the resistance to processes of cell deterioration gradually weakens. In detail, with developmental events taking place, cumulative physiological changes occur, such as the loss of water from the senescing tissue, leakage of ions, transport of metabolites to different tissues, and biochemical changes, such as the generation of ROS, increases in membrane fluidity and peroxidation, and hydrolysis of proteins, nucleic acids, lipids, and carbohydrates. These downward conditions belong to yin. Those factors with protective properties, such as chlorophyll content, chloroplast number, antioxidant enzyme activities, known as upward conditions, pertain to yang. ABA is a key regulator for keeping yin-yang coordination in plant life. The senescence conditions lead to cumulative ABA with age. Under these conditions without ABA, leaf cells undergo rather orderly changes in cell structure, metabolism, and gene expression. The earliest and most significant change in cell structure is the breakdown of the chloroplast and the other organelles. Metabolically, carbon assimilation is replaced by the catabolism of chlorophyll and macromolecules such as proteins, membrane lipids, and RNA. This could also explain why the application of exogenous ABA accelerates senescence in detached leaves, in which the high level of ABA is similar to that in stress conditions. Therefore, ABA is a factor controlling the onset of leaf senescence.

## Author contributions

YS, YM, CM, and CS designed the research. YS, FX, and GZ. performed the research. YS and CS wrote the article.

### Conflict of interest statement

The authors declare that the research was conducted in the absence of any commercial or financial relationships that could be construed as a potential conflict of interest.

## Supplementary material

Table S1 **List of primers used in this study**.

Table S2 **Complementation tests of Arabidopsis ***eas1*** and ***aba*** mutants**.

Figure S1 **EAS1 mutation accelerates plant senescence and flowering under unstressed conditions. (A)** Comparison of flowering phenotype from 40-day-old wild-type and *OE-EAS1* and *eas1* mutant plants grown under unstressed conditions in soil. **(B)** Thirty-five-day-old wild-type (Col-0 background) and *aba2-1* (Col-0 background) mutant plants grown under unstressed conditions. **(C)** Seedlings of wild-type and *OE-EAS1* and *eas1* mutant plants grown for 30 days on agar plate in unstressed growth conditions. *eas1-1* and *eas1-2* mutant plants show the senescence phenotype of yellowing leaves.

Figure S2 **The ***eas1*** mutants are two new ***aba2*** alleles. (A)** Positional cloning of *EAS1*. EAS1 was mapped to chromosome 1 between BAC clones F9I5 and F6D8. The *eas1-1* and *eas1-2* mutations were generated in the second exon in the *ABA2* gene (At1g52340). Closed boxes represent the open reading frame. The red dot is the centromere of chromosome 1. **(B)** ABA content of the fifth rosette leaf of wild-type and mutant plants at day 25 after sowing under unstressed condition in soil. **(C)** Germination of *eas1-1 and eas1-2* mutant seed is apparently insensitive to salt and mannitol treatment at different concentrations compared with wild-type seed. Seeds were germinated on agar medium supplemented with different concentration of NaCl or mannitol.The data collection were performed at day 5 after germination. **(D)** Infrared image of wild-type and *eas1-1* and *eas1-2* mutant plants at day 25 after sowing under unstressed conditions in soil. **(F)** Rate of water loss of detached leaves of wild-type and *eas1-1* and *eas1-2* mutant plants. Three experiments were performed with similar results. Error bars indicate standard deviations, while asterisks indicate significant differences from wild-type plants under Student's test (^**^*p* < 0.01).

Figure S3 **Determination of viability of wild-type and ***eas1***-***1*** mutant protoplasts in an age-dependent manner. (A,B)** Estimation of mesophyll protoplast activity by FDA staining. Bars indicate standard errors, while asterisks indicate significant differences from wild-type plants (^*^*p* < 0.05, ^**^*p* < 0.01); three experiments were performed with similar results. **(C)** Total protein extracted from the same weight of fresh leaf of the first to sixth true leaves of wild-type and *eas1-1* mutant plants. Ribulose 1,5-bisphosphate carboxylase/oxygenase (RBCL) is a protein marker. Proteins were visualized by coomassie blue staining.
