# Supplementary material for: Abscisic Acid as an Internal Integrator of Multiple Physiological Processes Modulates Leaf Senescence Onset in Arabidopsis thaliana
Source: Front Plant Sci. 2016 Feb 19;7:181. doi: 10.3389/fpls.2016.00181 (PMC4759271; doi:10.3389/fpls.2016.00181)
Supplement: Table S1 — List of primers used in this study. [file Table1.docx]

**Table S1.** List of primers used in this study.

| Code | 5' - 3' sequence | Use |
| --- | --- | --- |
| *EAS1-F(SalI)* | GCGAAGCTTATGTCAACGAACACTGAATC | overexpression vector |
| *EAS1-R (EcoRI)* | GCGGGATCCTCATCTGAAGACTTTAAAGG | overexpression vector |
| *CAB3-F* | GTTGCCAAGCCAAAGGGTC | qRT-PCR |
| *CAB3-R* | CTGAAAGTCCAGCGGTGTC | qRT-PCR |
| *RBCS-F* | CCGATTGGAAAGAAGAA | qRT-PCR |
| *RBCS-R* | TGCGGATAAGGTAGTCA | qRT-PCR |
| *SAG12-F* | TCTTCTGGTGCTTTGCC | qRT-PCR |
| *SAG12-R* | GCGTCTTCGCCTTTGTA | qRT-PCR |
| *SAG13-F* | TATGGAGCAACCAAAGGAGC | qRT-PCR |
| *SAG13-R* | GACAAAGAAATGCCACAAGC | qRT-PCR |
| *SAG14-F* | GGCAGTTGTATCAGAAGC | qRT-PCR |
| *SAG14-R* | GTGGTCCAGTGGTGTTTA | qRT-PCR |
| *SAG18 -F* | TGCGAGGTGAGAAAATAGGATG | qRT-PCR |
| *SAG18-R* | CAAGACCAGCAAGAAGTAAAGG | qRT-PCR |
| *SAG21-F* | ATCGTATCTGCTTTCGTCTC | qRT-PCR |
| *SAG21-R* | TCCACTCCCTTCTTCTTCAT | qRT-PCR |
| *SAG29-F* | GTTGTGACGGAATCGGTGGA | qRT-PCR |
| *SAG29-R* | ACGGTTTCAGGACGAGTAGC | qRT-PCR |
| *SAG101-F* | CTCCGAGAACAACCCATCA | qRT-PCR |
| *SAG101-R* | GCAACAGACCCTCCTAACG | qRT-PCR |
| *SAG113-F* | TGGTCGTGTTATCTACTGG | qRT-PCR |
| *SAG113-R* | GCTGATTACATACGGCTTC | qRT-PCR |
| *ACA3-F* | AGACGGCAGCCTGGTAAC | qRT-PCR |
| *ACA3-R* | AGTGCGAGGTGAGTAGCG | qRT-PCR |
| *CAX1-F* | TGCCATCTGTTTCTTCG | qRT-PCR |
| *CAX1-R* | GGGTGTTGTATGCCTCA | qRT-PCR |
| *CAX2-F* | CATTCTGCGTGGTGATTGGA | qRT-PCR |
| *CAX2-R* | ATGAACAGCATCGCCGTCTC | qRT-PCR |
| *CNGC1-F* | GCGGTGTTCATTTCTATTGC | qRT-PCR |
| *CNGC1-R* | CTTTACCCTCATCTCCTCCA | qRT-PCR |
| *TPC1-F* | GTTGATGTGCTGGTTGA | qRT-PCR |
| *TPC1-R* | CCGAAGTTCCCTTATGC | qRT-PCR |
| *PSBA-F* | AGAGAGACGCGAAAGCGAAAG | qRT-PCR |
| *PSBA-R* | GGATCATCAAAACACCAAACC | qRT-PCR |
| *PETB -F* | CTAGGCGGAATTACCCTTACT | qRT-PCR |
| *PETB -R* | GTCAATACACCCAGAACCACA | qRT-PCR |
| *Actin8-F* | TTCCTCATGCCATCCTCCGTCTT | qRT-PCR |
| *Actin8-R* | CAGCGATACCTGAGAACATAGTGG | qRT-PCR |
