# Supplementary material for: Abscisic Acid as an Internal Integrator of Multiple Physiological Processes Modulates Leaf Senescence Onset in Arabidopsis thaliana
Source: Front Plant Sci. 2016 Feb 19;7:181. doi: 10.3389/fpls.2016.00181 (PMC4759271; doi:10.3389/fpls.2016.00181)
Supplement: Table S2 — Complementation tests of Arabidopsis eas1 and aba mutants. [file Table2.docx]

**Table S2.** Complementation tests of Arabidopsis *eas1* and *aba* mutants

| Crosses | Generation | Total seeds | Mutant |
| --- | --- | --- | --- |
| *eas1-1 × eas1-2* | F1 | 182 | 182 |
| *eas1-1 × aba1-1* | F1 | 216 | 0 |
| *eas1-1 × aba2-1* | F1 | 197 | 197 |
| *eas1-1 × aba3-2* | F1 | 124 | 0 |
| *eas1-1 × nced3-1* | F1 | 138 | 0 |
