# Supplementary figures and images for: Abscisic Acid as an Internal Integrator of Multiple Physiological Processes Modulates Leaf Senescence Onset in Arabidopsis thaliana
Source: Front Plant Sci. 2016 Feb 19;7:181. doi: 10.3389/fpls.2016.00181 (PMC4759271; doi:10.3389/fpls.2016.00181)

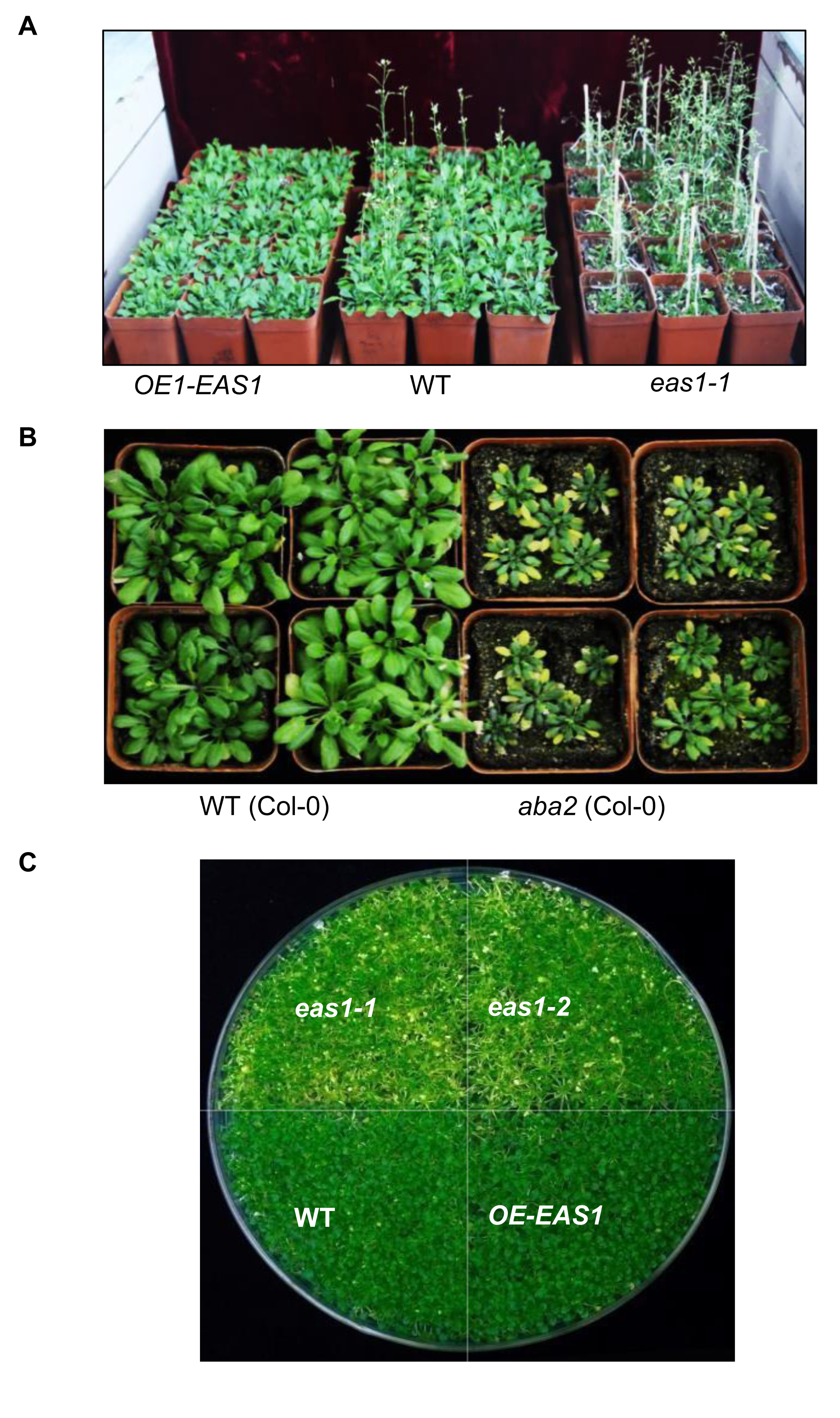

Supplement: Figure S1 — EAS1 mutation accelerates plant senescence and flowering under unstressed conditions. (A) Comparison of flowering phenotype from 40-day-old wild-type and OE-EAS1 and eas1 mutant plants grown under unstressed conditions in soil. (B) Thirty-five-day-old wild-type (Col-0 background) and aba2-1 (Col-0 background) mutant plants grown under unstressed conditions. (C) Seedlings of wild-type and OE-EAS1 and eas1 mutant plants grown for 30 days on agar plate in unstressed growth conditions. eas1-1 and eas1-2 mutant plants show the senescence phenotype of yellowing leaves. [file Image1.JPEG]

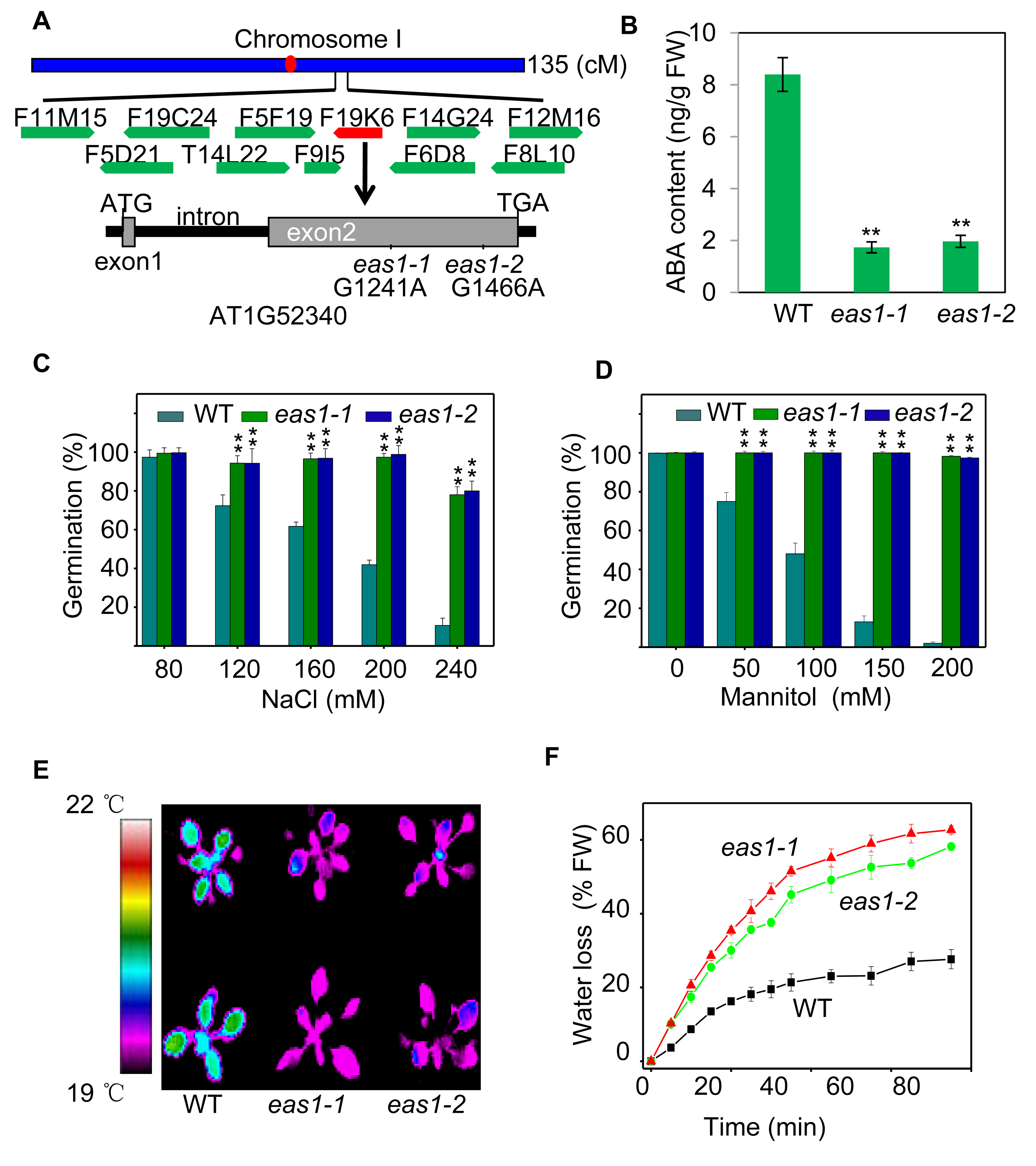

Supplement: Figure S2 — The eas1 mutants are two new aba2 alleles. (A) Positional cloning of EAS1. EAS1 was mapped to chromosome 1 between BAC clones F9I5 and F6D8. The eas1-1 and eas1-2 mutations were generated in the second exon in the ABA2 gene (At1g52340). Closed boxes represent the open reading frame. The red dot is the centromere of chromosome 1. (B) ABA content of the fifth rosette leaf of wild-type and mutant plants at day 25 after sowing under unstressed condition in soil. (C) Germination of eas1-1 and eas1-2 mutant seed is apparently insensitive to salt and mannitol treatment at different concentrations compared with wild-type seed. Seeds were germinated on agar medium supplemented with different concentration of NaCl or mannitol.The data collection were performed at day 5 after germination. (D) Infrared image of wild-type and eas1-1 and eas1-2 mutant plants at day 25 after sowing under unstressed conditions in soil. (F) Rate of water loss of detached leaves of wild-type and eas1-1 and eas1-2 mutant plants. Three experiments were performed with similar results. Error bars indicate standard deviations, while asterisks indicate significant differences from wild-type plants under Student's test (**p < 0.01). [file Image2.JPEG]

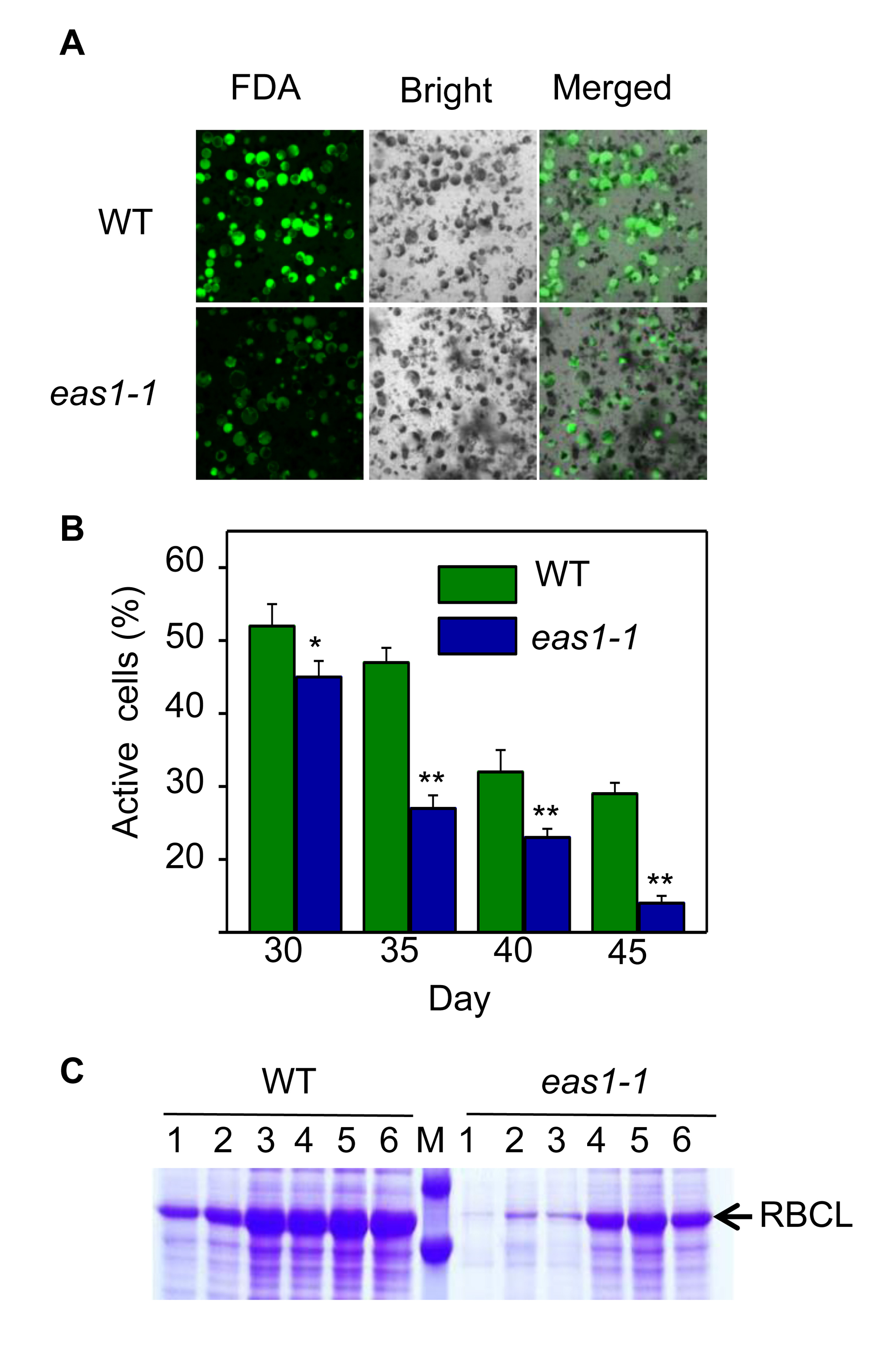

Supplement: Figure S3 — Determination of viability of wild-type and eas1-1 mutant protoplasts in an age-dependent manner. (A,B) Estimation of mesophyll protoplast activity by FDA staining. Bars indicate standard errors, while asterisks indicate significant differences from wild-type plants (*p < 0.05, **p < 0.01); three experiments were performed with similar results. (C) Total protein extracted from the same weight of fresh leaf of the first to sixth true leaves of wild-type and eas1-1 mutant plants. Ribulose 1,5-bisphosphate carboxylase/oxygenase (RBCL) is a protein marker. Proteins were visualized by coomassie blue staining. [file Image3.JPEG]
